# Phytosomes as an Emerging Nanotechnology Platform for the Topical Delivery of Bioactive Phytochemicals

**DOI:** 10.3390/pharmaceutics13091475

**Published:** 2021-09-15

**Authors:** Waleed S. Alharbi, Fahad A. Almughem, Alshaimaa M. Almehmady, Somayah J. Jarallah, Wijdan K. Alsharif, Nouf M. Alzahrani, Abdullah A. Alshehri

**Affiliations:** 1 Department of Pharmaceutics, Faculty of Pharmacy, King Abdulaziz University, P.O. Box 80260, Jeddah 21589, Saudi Arabia; wsmalharbi@kau.edu.sa (W.S.A.); amnalmehmady@kau.edu.sa (A.M.A.); 2 National Centre for Pharmaceutical Technology, Life Science and Environment Research Institute, King Abdulaziz City for Science and Technology (KACST), P.O. Box 6086, Riyadh 11442, Saudi Arabia; falmughem@kacst.edu.sa (F.A.A.); sjarallah@kacst.edu.sa (S.J.J.); walsharif@kacst.edu.sa (W.K.A.); nmalzahrani@kacst.edu.sa (N.M.A.)

**Keywords:** phytosomes, nanocarriers, skin barrier, phytochemicals, topical application, drug delivery

## Abstract

The emergence of phytosome nanotechnology has a potential impact in the field of drug delivery and could revolutionize the current state of topical bioactive phytochemicals delivery. The main challenge facing the translation of the therapeutic activity of phytochemicals to a clinical setting is the extremely low absorption rate and poor penetration across biological barriers (i.e., the skin). Phytosomes as lipid-based nanocarriers play a crucial function in the enhancement of pharmacokinetic and pharmacodynamic properties of herbal-originated polyphenolic compounds, and make this nanotechnology a promising tool for the development of new topical formulations. The implementation of this nanosized delivery system could enhance the penetration of phytochemicals across biological barriers due to their unique physiochemical characteristics, improving their bioavailability. In this review, we provide an outlook on the current knowledge of the biological barriers of phytoconstituents topical applications. The great potential of the emerging nanotechnology in the delivery of bioactive phytochemicals is reviewed, with particular focus on phytosomes as an innovative lipid-based nanocarrier. Additionally, we compared phytosomes with liposomes as the gold standard of lipid-based nanocarriers for the topical delivery of phytochemicals. Finally, the advantages of phytosomes in topical applications are discussed.

## 1. Introduction

### 1.1. Phytochemicals

Phytochemicals are bioactive polyphenolic compounds naturally found in plants that have been studied extensively due to their potential medicinal and nutritional benefits to humans. They not only play a protective role for the plant but are responsible for its color, aroma, and flavor. These compounds have attracted the attention of scientists worldwide, owing to their potent bioactivity against different diseases, their low cytotoxicity and their ability to be utilized in the production of cosmetics and dietary supplements [1,2,3].

Phytochemicals are categorized into three major categories based on their structural elements: terpenoids, alkaloids and polyphenolic substances. Numerous flavor and aromatic molecules are trepeniods, including menthol, linalool, geraniol, and caryophyllene, while catechols, lignins, tannins, stilbenes, and flavonoids are phenolic compounds. Alkaloids are further divided into Pyrrolidine, Pyrrolidine-pyridine, Pyridine-piperidine, and Isoquinoline alkaloids based on their heterocyclic ring systems [4]. There are a variety of ways in which phytochemicals can exhibit their influence, including acting as substrates, cofactors, or inhibitors of enzymatic reactions [5,6,7]. By demonstrating their chelating properties, they can remove undesirable constituents from the gastrointestinal tract. Additionally, phytochemicals can be utilized to enhance the uptake and stability of a variety of essential nutrients [8]. The antioxidant activity of phytochemicals is one of their potential properties that has an important function in scavenging free radicals in human tissue [9]. 

An example of the utility of phytochemicals is salicin, which is a compound found in willow tree bark. It has anti-inflammatory and analgesic activity, and is also used as a precursor to numerous conventional non-steroidal anti-inflammatory drugs (NSAIDs). Due to its widespread use and high demand, greater quantities of salicin were prepared using chemical synthesis [10]. Certain phytochemicals also have nutritional and energetic properties, which is why they are referred to as nutraceuticals [7,11]. Efforts have been made to identify bioactivities in different phytochemicals compounds extracted from a variety of plant species that can be implemented in the treatment of different diseases. Despite the potential bioactivity of phytochemicals, there remain concerns about their quality, safety and efficacy [12].

Nowadays, the skin is more likely to become infected as a result of changing environmental conditions and an increase in pollution levels. Consequently, there has been a significant increase in the demand for herbal medicine in both developed and developing countries owing to their potent biological efficacy, higher safety margins, and lower cost than synthetic agents [13]. Numerous herbal extracts originating from different plant species have been evaluated for their potential to treat skin conditions due to their medicinal benefits, which include antimicrobial and anti-inflammatory properties, their ability to promote blood clotting and wound healing, and to relieve burns and other skin diseases [14,15]. Several common skin conditions, including eczema, acne, urticaria, pruritus, psoriasis, and other bacterial and fungal skin diseases, can be treated efficiently using medicinal herbs [16,17]. The chemical structure of most plant extracts used in pharmaceutical and cosmeceutical applications is based on flavonoids or other polyphenol rings, which have high molecular weight and, hence, have poor solubility and are poorly absorbed through the skin [18,19]. 

### 1.2. Structural Nature and Barrier Properties of the Skin 

**Figure 1:**
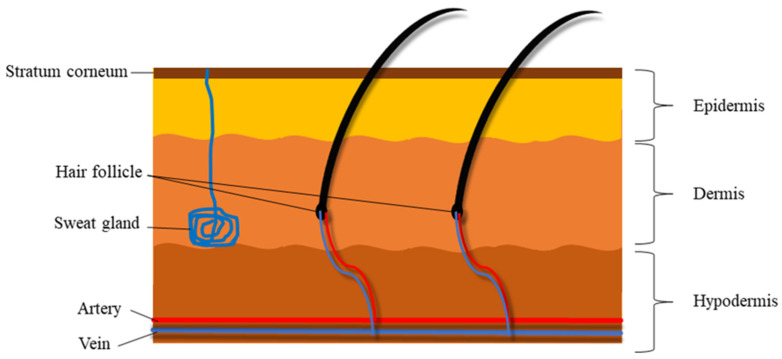
Simplified structure of the skin and its barriers.

The human skin is a remarkably complex and sophisticated organ that serves as a barrier to external exposure by enveloping the inner body components. It consists of three main layers—the epidermis, dermis and hypodermis—and each has different degrees of specialization. Along with its function as a waterproof barrier, the epidermis contains melanocytes, which determine the skin’s tone and pigmentation. The dermis is the skin layer underneath the epidermis that composed of connective tissue, sweat glands and hair follicles and helps to maintain the skin’s flexibility. In comparison to the other layers of the skin, the hypodermis is made up of fat and connective tissue and is responsible for shock absorption [20]. The stratum corneum (SC), or outer layer of the epidermis, is entirely made up of dead, keratinized epithelium cells and other metabolically inactive cells that cover the outer surface of the skin [21]. The SC and epidermis are regarded as the most effective barriers against hydrophilic and lipophilic compounds, respectively [22,23]. Furthermore, in terms of skin mechanical properties, elastic fibres are essential for skin elasticity and are directly responsible for skin performance and appearance [24]. Figure 1 shows a simplified structure of the skin and its barriers.

**Figure 2:**
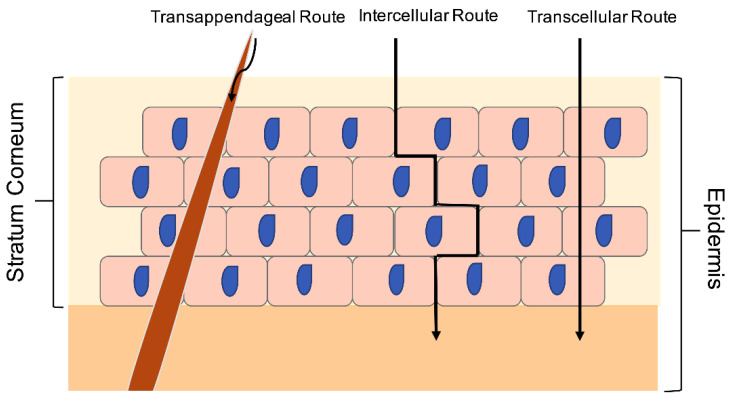
Mechanism of transdermal drug delivery.

In terms of skin permeation, there are three possible cellular routes for active compounds penetration across the SC: transcellular, intercellular, and appendageal diffusion (i.e., sweat glands and hair follicles) [25]. The latter route is, however, considered to be insignificant due to the appendages occupying only a relatively low surface area (e.g., approximately 0.1% of the forearm skin area) [26]. The main route of the compound’s penetration across the SC is via the intracellular pathway, which causes a direct penetration of the SC to reach the lower layer of the epidermis or even lower to the dermis layer [26]. Figure 2 shows the mechanism of transdermal drug delivery. 

**Figure 3:**
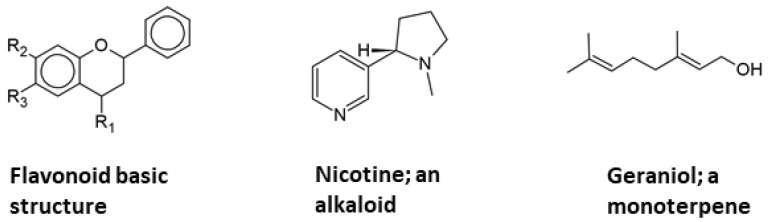
The chemical structure of major classes of phytochemicals.

The skin is an excellent site for the administration of pharmaceutical agents for both local and systemic impacts and serves as a strong barrier to the permeability of the majority of substances [27]. The transdermal permeability can be increased when the active ingredients are lipophilic and have a low molecular weight. Most of the widely studied phytochemicals are polyphenols-based compounds such as flavonoids, alkaloids, and terpene, as shown in Figure 3, which have poor bioavailability and low lipid solubility due to their hydrophilic nature, which limits their in vivo activity [28]. The multiple ring structures of polyphenols results in large particle size compounds being unable to cross the biological membrane by passive diffusion [29]. Many flavonoids, such as glycyrrhizic acid and silymarin, have significant value in medicine and cosmetics when applied topically. Nevertheless, their topical application is restricted due to poor absorption through the skin structure [30]. Absorption through the skin is influenced by a number of factors such as skin temperature, hydration, and the permeability of ingredients due to molecular size, which is believed to be the most important factor affecting dermal penetration [21,31]. Furthermore, particle-related factors, such as surface properties, coating presence, and surface charge type, can affect skin penetration [32]. Therefore, the loading of these herbal polyphenol phytochemicals in a novel delivery system can facilitate their penetration across the skin barriers and would be essential to enhance their topical bioavailability [33].

### 1.3. Nanotechnology Platform for the Delivery of Bioactive Phytochemicals 

As mentioned previously, the chemical structure for most of the phytochemicals in the plant kingdom that have biological activity and can be utilized in the pharmaceutical and cosmeceutical fields are mainly based on polyphenolic compounds [34], which have a multiple-ring structure, high miscibility in the aqueous phase, and high molecular weight [29,35]. These bioactive polyphenolic compounds are formed in the plant as secondary metabolites to perform different functions, and are widely present and easily extracted from a variety of plant species [36]. The outstanding properties of polyphenolic compounds have attracted researchers in the field of pharmaceutical science owing to their potent therapeutic efficacy against different diseases and their capacity to be utilized in the production of cosmetics and dietary supplements [37]. Several studies demonstrated that polyphenolic phytochemical-rich products have potent anti-inflammatory, anticancer, antidiabetic, and antioxidant activities in addition to other multiple health benefits to human organs [9,38,39,40]. The chemical nature of these components has a crucial impact on the lipid solubility properties, as poor penetration across lipid-rich biological membranes, such as the skin barrier, has been reported, which leads to poor bioavailability [18]. This is considered the main challenge regarding the translation of phytochemicals’ therapeutic activity to clinical settings [41]. 

To overcome the low absorption of bioactive polyphenolic phytocompounds, nanosized drug delivery systems can be utilized to enhance their penetration across biological barriers thanks to their unique physicochemical properties, increasing the bioavailability of phytocompounds [42,43,44]. Nanocarriers have an important function over conventional phytochemicals obtained at other scales in protecting the bioactive polyphenolic phytochemicals from oxidation and degradation, hence maintaining their long-term effects as well as improving their stability [45,46]. The high surface area of nanoparticles supports the effective delivery of active ingredients into the skin in the case of topical applications of phytochemicals [47]. The mechanism of polyphenolic phytochemicals’ incorporation into nanoparticles is by the hydrogen bond (H-bond) or through hydrophobic interactions [45]. 

There are various types of nanotechnology-based materials that are currently used in the delivery of polyphenolic phytochemicals. Nanosized delivery systems can be classified into two main groups: Organic delivery systems (i.e., liposomes and polymeric nanoparticles) and inorganic delivery systems (i.e., silver, gold, and copper nanoparticles) [48]. Liposomes are one of the most commonly used nanoparticles that have been successfully used in the pharmaceutical and cosmetics fields [49]. The encapsulation of curcumin into liposomal nanoparticles showed potent activity against lung, pancreatic, and colorectal cancer at a lower dose in comparison to free curcumin [50]. Polymeric nanoparticles can also be used as an efficient nanocarrier of phytochemicals, and the encapsulation of curcumin extract into chitosan and polylactic-co-glycolic acid (PLGA) nanoparticles exhibited a significant improvement in its solubility profile compared to a conventional curcumin formulation [51]. 

Metallic inorganic nanoparticles, such as silver, have also been used as a nanocarrier for phytocompounds. Ginseng herbal extract showed stronger anticancer activity in vitro against A549 cells at a lower dose when formulated as silver nanoparticles [52]. Using gold nanoparticles as a delivery system of *Moringa oleifera* demonstrated potent therapeutic activity against the in vitro model of non-small cell lung carcinoma [53]. Sankar et al. studied the effect of *Ficus religiosa* incorporation into copper nanoparticles and the results showed a significant increase in anticancer activity [54].

Another example of how nanoparticulate delivery platforms can be used to overcome SC barriers is transferosomes [55]. Transferosomes can transport hydrophilic substances and biomacromolecules into deeper layers of the epidermis, resulting in a sustained release effect and improved bioavailability [56]. This type of lipid nanocarriers is more elastic, ultra-deformable, and adaptable to external stress than liposomes (inflexible lipid layer) or niosomes (non-ionic surfactant-based vesicles) [56]. Four essential components make up transferosomes: phospholipids, an edge activator (surfactant or bile salt), ethanol, and water, which serves as the vehicle for transport. An edge activator is required to destabilize the lipid bilayer and increase membrane deformability. Transferosomes can spontaneously squeeze through SC channels to avoid vesicle rupture when passing through numerous skin layers [57]. *Curcuma longa* extract, capsaicin, vincristine, and colchicine are examples of natural compounds that can be formulated using transferosomes as a delivery vehicle [58,59,60,61].

In recent years, there have been significant developments of novel nanosized delivery systems that may enhance the absorption and penetration of herbal bioactive materials through biological membranes, and therefore increase their bioavailability [62]. One of these emerging nanotechnologies that can be implemented to overcome the poor bioavailability of bioactive phytoconstituents and enhance their miscibility in lipid-rich barriers is phytosomes [63].

## 2. Phytosomes

### 2.1. Phytosome Significance in Drug Delivery

Phytosomes are an innovative lipid-based delivery system that have a liposomes-related structure and can be used for the entrapment of different types of polyphenolic-based phytoconstituents to improve their absorption when administrated [63,64,65]. The first phytosomes were developed by Indena company (Milan, Italy) in the late 1980s, which aimed to increase the bioavailability of drugs by complexing them to phospholipids. The structure of phytosomes is composed of standardized polyphenolic plant extract incorporated into phospholipids, mainly phosphatidylcholine (PC) [28]. The lipid vesicles of phytosomes are the result of a H-bond interaction between the polyphenolic moiety of the bioactive herbal extracts and the phosphate group of phospholipids matrix in non-polar solvents [66]. The water-soluble polyphenolic rings of phytochemicals (i.e., flavonoids and terpenoids) have a high affinity to chemically bind to the hydrophilic moiety of phospholipids (i.e., choline) to form the body of phytosomes, while the phosphatidyl lipophilic moiety of the phospholipids forms a tail to incorporate the water-soluble choline-bound phytoconstituents [67,68]. 

The encapsulation of poorly soluble polyphenolic compounds into the phytosomal delivery system has a significant effect on the enhancement of their absorption, leading to better penetration and absorption across the biological membrane and enhanced bioavailability [69]. The bifunctional nature of phytosomes has been demonstrated to improve their pharmacodynamic and pharmacokinetic properties in comparison with conventional herbal compounds when applied topically, owing to their capabilities in the transition between lipophilic and hydrophilic barriers of the skin [28,70]. The potential role of phytosomes in the improvement of herbal-originated polyphenolic compounds used for the treatment of several diseases makes this nanotechnology a promising tool for the development of new formulations. 

Phytosomes are usually prepared by mixing the active biological phytoconstituents with phospholipids, such as PC, phosphatidylserine (PS), and phosphatidylethanolamine (PE), in specific stoichiometric ratios under certain conditions [71]. Following mixing, aprotic solvents, such as ethyl acetate, methylene chloride, dioxane and acetone, are evaporated under a constant vacuum to isolate the complex completely, meaning the phytoconstituents will be incorporated into the phytosomes lipid vesicles [72]. 

The emergence of phytosomes nanotechnology has a potential impact in the field of drug delivery to resolve the barriers of poor lipid solubility and improve the bioavailability of bioactive phytochemicals such as silybin, ginkgo and polyphenolic compounds found in olive oil. Several phytosomes-based products are being formulated and commercialized, some of which are described in Table 1. Silybin, the active ingredient of *Silybum marianum* (milk thistle), is a water-soluble flavonoid reported to have potent hepatoprotective and antioxidant activities [73]. However, silybin has poor solubility and absorption in a lipid-rich biological membrane. The formulation of the milk thistle extract as a phytosome delivery system increased its absorption and led to a sevenfold increase in antioxidant activity in comparison to free silybin [74]. Moreover, oral administration of silybin-phytosome preparation showed remarkable enhancement in bioavailability in rats [75]. 

Using phytosomes nanotechnology for the delivery of ginkgo herbal extract had beneficial results in the pharmacokinetic profile and enhanced brain and vascular protection [76]. A study conducted with human volunteers demonstrated that the incorporation of *Gingko biloba* extract into a phytosomes delivery system increased the absorption of its flavonoids and terpenes constituents significantly in comparison to the free extract [29]. Oleaselect is a commercialized product based on the polyphenols of olive oil and is manufactured as a phytosomes formulation. Anti-inflammatory, antioxidant, and antihyperlipidemic activity, as well as cardiovascular protection, were reported to be enhanced when it was administrated as a phytosomal formulation in comparison to standard oil [77]. The preparation of *Centella asiatica* in the form of phytosomes was found to reduce the oxidative stress in diabetic patients and enhance protection against ischemic-reperfusion damage in rat heart [78]. Greenselect herbal extracts exerted several benefits, such as the ability to scavenge free radicals, antioxidant activity, and proinflammatory cytokine formation interference, among others. The formulation of Greenselect as phytosomes was reported to improve the bioavailability of this extract and, therefore, its bioactivity [79]. 

The phytosomal formulation of rutin showed significantly higher dermal absorption when compared to the conventional rutin formulation, resulting in greater activity against rheumatoid arthritis [80]. The incorporation of poorly soluble curcumin into a phytosomes delivery system enhanced hepatic protection via the restoration of liver glutathione system [81]. The bounding of the flavonoids in the bioactive curcumin with the phosphatidylcholine moiety of 1,2-dimyristoyl-sn-glycero-3-phosphocholine facilitated the absorption of curcumin in rats in comparison with the standard composition [82]. The therapeutic activity of other phytoconstituents, such as *Vitis vinifera* [28], *Echinacea augustifolia* [83], *Cartaegus mexicana* [84], *Carteagus species* [65] and *Ruscus aculeatus* [85], has been shown to be significantly improved when incorporated into phytosomes.

### 2.2. Phytosomes in Clinical Trials

Following the phase of preclinical studies, several phytosome-based formulations have reached the clinical trial in order to perform further investigations on drug saftey and how a drug can interact with human body. This is very crucial step toward obtaining the final approval from FDA. In 2007, the first clinical trial on phytosome-based formulation was conducted (ClinicalTrials.gov Identifier: NCT00487721, accessed date: 28 August 2021). Silybin with its reported anti-cancer activity [86] was incorporated into phytosomes to be used in prostate cancer patients before their prostatectomy. The preliminary result demonstrated that a high oral dose of silybin-phytosome achieves transient high blood concentration, and concluded that this phytosomal formulation could be used as an alternative future therapy in the treatment of patients with prostate cancer [87,88]. The second study that reached clinical trial was also based on silybin-phytosome formulation (ClinicalTrials.gov Identifier: NCT02146118, accessed date: 28 August 2021). The phytosomal formulation was used as combination therapy with Erlotinib (Tarceva) and investigated under the name of Siliphos. This study is still under investigation to date, and they proposed that this combination could have synergistic effect in the treatment of patients With EGFR mutant lung adenocarcinoma. In 2014, a study on the efficacy of green tea extract loaded into phytosome on obesity was conducted (ClinicalTrials.gov Identifier: NCT02542449, accessed date: 28 August 2021). The administrated formulation was used to control the weight in obese patients following their weight loss, and this study is currently in phase IV in clinical trial. The result showed a significant effect of green tea extract phytosomal preparation in maintaining the weight in obese patients following their weight loss^®^ [89]. Grape seed extract was also investigated in clinical trial to explore its efficacy against early stages lung cancer when prepared as phytosome-based formulation (ClinicalTrials.gov Identifier: NCT04515004, accessed date: 28 August 2021). The study outcome exhibited that the phytosomal formulation delayed the planned surgery of >14 days. The activity of bergamot-phytosome preparation as anti-hypercholesterolemic agent in subjects with mild hypercholesterolemia when combined artichoke leaf dry extract was studied (ClinicalTrials.gov Identifier: NCT04697121, accessed date: 28 August 2021). The result showed that the administration of prepared formulation has positive impact on lipid and metabolic parameters, hence significant activity as anti-hypercholesterolemic agent was achieved. The most recent study reached clinical trial has explored the adjuvant benefits of quercetin phytosome in treatment of patients with COVID-19 (ClinicalTrials.gov Identifier: NCT04578158, accessed date: 28 August 2021). It is proposed that quercetin phytosome will contribute to boosting the natural immunity of the subjects and will help in preventing the COVID-19 disease progression (i.e., preventing the need of hospitalization). The study is still under investigation to date. Table 2 summarizes the phytosome-based formulations on clinical trials. 

### 2.3. Phytosomes and Other Lipid-Based Nanocarriers: Similarities and Differences

In the field of topical dosage form, the optimal pharmaceutical vehicle should facilitate the penetration of incorporated molecules efficiently through the skin barriers, protect the loaded compounds from degradation, be easy to prepare, be safe for the skin, and not cause dermal sensitivity or irritation [26,90]. Phytosomes have been demonstrated to effectively deliver poorly soluble molecules via the incorporation of these materials in the lipid bilayer membrane or through conjugation with the lipid composition [91]. To evaluate phytosomes as an emerging nanotechnology for topical applications and to determine their capability to transport biomaterials efficiently through the skin barriers, they need to be compared with other lipid-based drug vehicles that proved to have superior characteristics in topical applications. 

**Figure 4:**
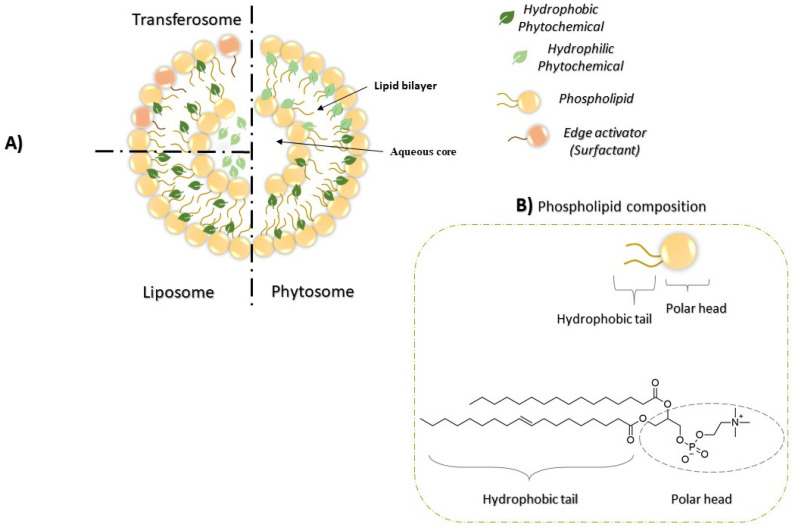
(**A**) Figurative representation of structural comparison between different lipid-based drug delivery systems (phytosomes, liposomes, and transferosomes). In the preparation step of phytosomes, phytochemicals are mixed with phospholipids to make phytochemical-phospholipid complex. This complex is mixed with cholesterol in suitable solvent to make phytosomes [91]. In case of liposomes and transferosomes preparation, phospholipid is mixed first with lipids to make the liposomal vesicle. Then, the phytochemical is incorporated in the liposome later [97,98]. (**B**) Represent the composition of phospholipid moiety. The phospholipid is composed of a polar head (which contains phosphate group) and hydrophobic tail (which composed of fatty acid chains). The most common example of polar head is PC. The chemical structure in the figure is the typical structure of the phospholipid derived from the soybean.

Liposomes and transferosomes are one of the most widely used lipid-based vehicles in the field of drug delivery nanotechnology for topical applications to facilitate the penetration of water-soluble compounds across the skin [92,93]. Phytosomes, liposomes and transferosomes are examples of lipid-based delivery systems that have the ability to encapsulate bioactive phytochemicals in order to increase the concentration of poorly soluble molecules, their absorption, and stability [94]. Since their discovery, different phytosomal and liposomal products for skin care have been approved and marketed, while very few transferosomal formulations have been translated into clinical products. Due to the structural similarities, as shown in Figure 4A, the comparison between these lipid-based drug delivery systems would be essential. 

Phytosomes retain some crucial characteristics of liposomes and transferosomes such as the ability to increase the solubility of poorly soluble molecules as in polyphenolic phytochemicals. Interestingly, phytosomes and transferosomes exhibit additional novel natural properties in the topical applications such as long-term stability and higher skin penetration [91]. These characteristics encouraged researchers to explore the similarities and differences between phytosomes and commonly used lipid-based nanocarriers (i.e., liposomes and transferosomes). The comparison should be established according to several criteria relevant to the area of nanotechnology such as the structure of lipid vesicles, their phospholipid composition, and the method of preparation. A good understanding of phytosomes’ special properties has crucial consequences in the field of topical dosage form industry and may improve the quality of topical products. 

Phytosomes have many similarities to liposomes and transerosomes in terms of structural and functional properties. Different types of lipids can be used to formulate the lipid bilayer structure of phytosomes such as cholesterol, PC, PS, PE, and glycosphingolipids [28]. Figure 4B shows the composition of phospholipid moiety of phytosomes which is composed of a polar head containing a phosphate group and hydrophobic tail composed of fatty acid chains. These lipids, in addition to others, are commonly used in the preparation of liposomes and transferosomes delivery systems. In liposomes, several cationic lipids such as 1,2-dioleoyl-3-trimethylammonium-propane (DOTAP), 1,2-distearoyl-sn-glycero-3-phosphoethanolamine (DSPE), and 3ß-[N-(N’,N’-dimethylaminoethane)-carbamoyl]cholesterol (DC-Chol) are commonly added to the formulation to improve their ability to cross the negatively charged cell membrane [95]. Transferosomes are mainly composed of phospholipids, an edge activator (i.e., surfactant or bile salt ranging from 10–25%), low percentage of ethanol, and water as a vehicle as shown in Figure 4A [56,57,96].

In terms of colloidal stability, phytosomes and transferosomes exhibited a greater physical stability in aqueous media than liposomes with no sign of aggregation in 4 °C and 25 °C up to three months [99,100], whereas liposomes should be freeze dried to maintain their stability. Different bioactive substances, including herbal products, can be packaged into these lipid-based nanocarriers and applied to the skin to facilitate their dermal absorption. In topical products, phytosomes and transferosomes are considered superior to liposomes in the delivery of active ingredients, owing to their higher absorption properties through the skin [101]. When compared with liposomes as the most commonly used drug delivery system for skin purposes, phytosomes and transferosomes demonstrated superior SC penetration resulting in high accumulation in the deeper areas of the skin (i.e., the epidermis and dermis). The high dermal penetration properties of transferosomes are owed to their ultra-deformability and the ability to squeeze via SC and transport as intact vesicles when the hydrodynamic diameter is below 300 nm [102,103]. The edge activator in transferosomes play an essential function in providing a high radius of curvature that leads to destabilize the lipid bilayer and increase membrane deformability, hence, the capability of transferosomes to squeeze through different skin layers can be observed [103].

Structurally, bioactive molecules are part of the phytosomes membrane [97], whereas the hydrophilic molecules are engulfed in the aqueous core of liposomes and transferosomes or entrapped in the lipid layer membrane if they have a lipophilic nature [98]. The structure of phytosomes is based on the H-bond interaction between the phytoconstituents and the polar moiety of phospholipid molecules [104], whereas no chemical interaction occurs in liposomes and transferosomes because the encapsulated materials are entrapped naturally in the lipid bilayer membrane or in the aqueous core [105]. The stability of incorporated compounds is much higher in phytosomes, owing to the chemical interaction, whereas one of the main challenges in liposomes nanotechnology is the leakage of bioactive molecules from the lipid-vesicles over time [106]. 

Phytosomes are typically prepared following dissimilar protocols of liposomes and transferosomes. Liposomes and transerosomes are usually formulated by thin hydration method [107,108]. Briefly, appropriate amounts of lipid mixture were dissolved in the suitable organic solvent and mixed in a round bottom flask. The lipid mixture then dried by nitrogen gas until the formation of thin film. Then, a hydration solvent (i.e., distilled water or phosphate buffer saline) containing the loaded material is added to the lipid thin film to form the multilamellar vesicles (MLV). These vesicles are then converted to unilamellar vesicles (ULV) using a sonicator or extruder [109]. However, the encapsulation method used for phytosomes mainly depends on the chemical conjugation between the phospholipid’s moiety and the polyphenol-based bioactive phytoconstituents [110]. 

Table 3 summarizes the key similarities and differences between different lipid-based drug delivery systems that commonly used in the topical delivery of herbal substances.

## 3. Advantages of Phytosomes in Topical Applications

In topical applications, phytosomes have several potential advantages over the conventional topical formulations. Phytosomes increase skin absorption and bioavailability, and they induce the delivery of herbal active constituents to tissues [111]. Moreover, phytosomes improve skin functions by enhancing hydration, the enzyme balance, and collagen structure [30]. The high affinity of phytosomes to skin phospholipids intensified its effectiveness compared to conventional free compounds [112]. As mentioned earlier, there are several barriers facing topical applications of phytosomes formulation. For example, one of the most important barriers of the transdermal application of phytochemicals is the SC, which is the thick outer layer of the epidermis [113]. Bioactive molecules can cross the SC via different pathways, which are either intercellular or intracellular. Intercellular penetration can be achieved via sweat glands, sebaceous, or hair follicles, whereas the intercellular lipid matrix and corneocytes are the main pathways of intracellular penetration [114,115]. It has been reported that enhancing the diffusion coefficient of the drug can increase the concentration of biomolecules and enhance partitioning between the these molecules and the SC layer, and that all these factors can improve the permeability of biomolecules to the SC for transdermal application [26]. The transdermal permeability of topical products can be increased when the active ingredients are lipophilic and have a low molecular weight. 

Most of the widely studied phytocompounds are polyphenols, which have poor bioavailability and lipid solubility due to their hydrophilic nature, limiting their in vivo activity [28]. The phospholipid moieties of phytosomes have a high affinity to bind several flavonoids compounds tightly [116]. There are several herbal extracts, such as hawthorn, grape seed, green tea, milk thistle and ginseng, that are more effective when they are loaded into phytosomes, even more so than when they are carried in a liposomal formulation [112]. The formulation of polyphenol-based phytochemicals phytosomes nanoparticles enhances the application of standard herbal materials as the phospholipid molecules of phytosomes interact with the active phytoconstituents, increasing their stability [117]. Furthermore, the phytosomes–herbal complex has a higher affinity to the skin phospholipid moiety, which can improve the lipid solubility of the topical formulation [112]. 

**Figure 5:**
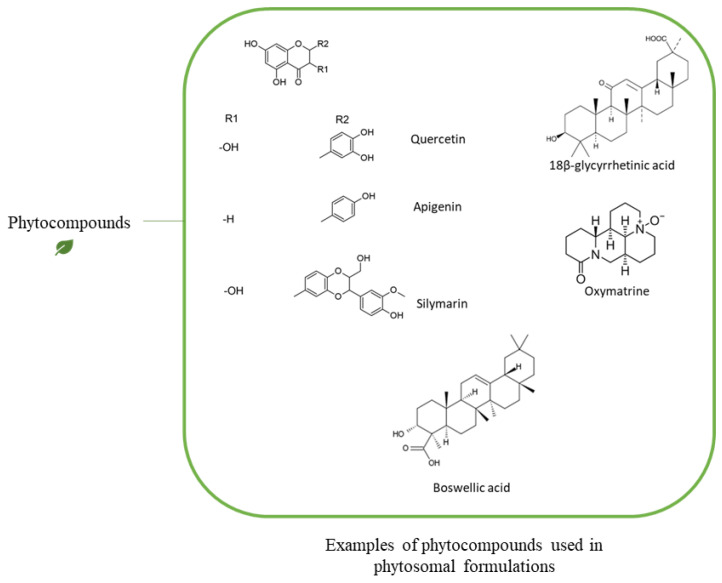
Scheme demonstrating examples of phytocompounds incorporated into a phytosomal delivery system.

Figure 5 demonstrates some examples of the phytocompounds incorporated into a phytosomal delivery system.

*G. biloba* extract has been used for different applications and can be applied topically for its antioxidant and antiaging agents [118]. There are various reports that have compared standard *G. biloba* extract and the extract complexed with phospholipids. It was reported that the topical application of *G. biloba* improved peripheral circulation due to its topical anti-inflammatory activity, and it was more effective in a complex with phospholipid moieties of phytosomes [119]. The bioavailability and pharmacokinetics profiles of the conventional *G. biloba* extract and the phytosomal form were evaluated by Chen et al., who showed that the bioavailability of the herbal extract increased significantly in the phytosomal complex [120]. In a clinical study by Kennedy et al., they compared the cognitive and mood effects of a low dose of *G. biloba* extract and products complexing the extract with two types of phospholipids (PS or PC) in human subjects. Their findings demonstrated that all treatments were associated with improved calmness; however, there was a modest enhancement in the therapeutic benefit of secondary memory performance for *G. biloba* extract complexed with PC [121].

Quercetin is a phenolic phytochemical compound found in various vegetables, fruits, and leaves, and has soothing antioxidant and anti-itching effects [122]. In a study by Maramaldi et al., the formulation of quercetin complexed with phytosomes nanoparticles exhibited potent dermal activity above that of standard quercetin and similar to the conventional anti-inflammatory drugs that are usually used [122]. The quercetin-phytosomes complex had a significant impact by reducing redness, itching, and inflammation of damaged skin. Research also suggested that this complex may also support restoration of the skin barrier function, increasing hydration, and reducing water loss [122]. In another report by El-Fattah et al., quercetin phytosomes demonstrated superiority in the dermal therapeutic benefit over free quercetin in an ovariectomized rat model [123]. Lycopene is a terpene molecule found in fruits and vegetables that is known for its antioxidant, antiproliferative, and anti-inflammatory activities. Its incorporation into lipid nanocarriers enhanced the dermal absorption significantly to tackle skin aging and other skin conditions [124,125]. Moreover, it can also produce anti-proliferative effect against tumour [126].

Molecular interaction of phytochemical-phospholipid complex has been studied using various analysis methods. Studies using 13C-NMR, 1H-NMR, 31P-NMR, IR, DSC, and X-ray revealed that complex formation resulted in chemical shifts and signal changes different from their original components [33,85,127,128,129,130,131]. All these studies confirm the generation of chemical bonds between the phospholipid’s moieties and phytochemicals moieties. Chemical interactions include H-bonding, van der waals interaction, and hydrophobic effect, dipole-dipole interaction [132]. The chemical structure of the phytochemicals can significantly affect the type of bonds formed during complex formation. For instance, lycopene, a type of hydrophobic β -carotenoids, consists of tetraterpene carbon chain interacts mainly via hydrophobic effect with the acyl chain of the phospholipid (i.e., fatty acid chain moiety and hydrophobic tail). This interaction has been confirmed by x-ray study where the signal of hydrophobic part of PC disappeared completely among the formation of the complex [133]. Quercetin, a polyphenolic compound, on the other hand, has a cyclic hydrophobic part and hydroxyl groups which makes it an amphiphilic compound. Studies have shown that quercetin mainly interacts via H-bonding with the polar head group and with a lesser extent with the acyl chain moiety of the phospholipid [127,128]. Saponins, are glycoside compounds consists of a parent compound (such as terpene) and sugar derivative. A study of saponins formed from pentacyclic triterpenes and one or more sugar units showed that saponin interacts with the polar head group of the phospholipid during complex formation [15]. Polyphenolic compounds extracted from olive oil fruit (i.e., tyrosol, verbascoside, hydroxytyrosol), interact mainly with the polar head group via H-bonding [33]. 18-β-glycyrrhetinic acid, a steroid like structure, contains pentacyclic rings and an acid group. The polar group of 18-β-glycyrrhetinic acid interacts via H-bond with the polar head group of phospholipids and formed a complex [132,134]. In the DSC thermogram, the complex of 18-β-glycyrrhetinic acid-phospholipid showed a signal different than that of 18-β-glycyrrhetinic acid alone or the phospholipid alone. For example, 18-β-glycyrrhetinic Acid (18β-GA) showed a sharp endothermic peak at 294° which revealed its melting and thermogram of phospholipids showed mild and broad endothermic peak at 277°, 203° and 96°, while thermogram of 18β-GA phytosome revealed endothermic peak at 245°, 198° and 80° [130]. Table 4 summarizes the main types of chemical interactions between phospholipids and phytochemicals.

In vitro skin permeation studies have shown better permeation parameters and higher permeability rate into skin when phytochemicals complexed with PC. For example, oxymatrine (OXM) when complexed with PC (OXM-PC) in microemulsion formulation demonstrated better flux (*Jss*) and permeability coefficient (*Kp*) compared to control, which is free oxymatrine solution (*Jss* was 253.63 ± 8.62 and 67.87 ± 8.03 µg/cm^2^.h for complexed and free formula, respectively) when complexed with PC [135]. Oxymatrine is a cyclic water-soluble compound found in a number of Chinese herbs, such as *Sophora flavescens*, *Sophora macrocarpa*, *Ammothamnus lehmannii*, *Euchresta horsfieldii* and *Leguminosae*, and can be used as anti-inflammatory agent [136]. 

Another example is boswellic acid (BA), which is the active phytoconstituent in *Boswellia serrata* extract. The extract has been widely used in the treatment of inflammatory conditions and has some cosmetic applications [137]. In vitro permeation studies on Caco-2 cell model of *B. serrata* extract complexed with phospholipid showed superior mass flux (J) than the free extract alone (J was 24.02 ± 2.08 and 3.07 ± 0.09 ng/ cm^2^.min, for complexed and free formula, respectively) [138]. 

As mentioned earlier, SC is a barrier for drugs and chemicals to penetrate to the skin and deliver topically. In a study by F.-H. Cao et al., where in vivo skin permeation studies in mice have shown that percent of OXM-PC complex retention in destartum corneum skins is higher than that of free oxymatrine solution [135]. The percent of retention ratio of OXM-PC reached a peak after 6 h (31.41%) and maintained a level of 22.37% after 24 h, while free (OXM) solution reached a peak after 9 h (17.23%) and decreased quickly to 4.56% after 24 h. This data confirmed that complexation with phospholipid can increase accumulation of OXM and enhance topical activity [135]. 

Moreover, phytosomes was found to release the phytoconstituent in higher percent than other vesicular systems such as liposomes and niosomes. In a study by Sharma et al. investigated the effect of BA phytosomes in producing topical anti-inflammatory effect in induced paw edema in rats compared to BA liposomes and BA niosomes. BA phytosomes were found to be the most in reducing the inflammation and edema after 1, 3, 5 h of topical application (78.26 ± 3.67, 89.23 ± 3.11, and 88.89 ± 3.17%), while BA liposomes and BA niosomes could reduce the inflammation after topical application, but it was less than the effect of BA phytosomes (52.17 ± 2.14, 80.00 ± 3.19, 77.78 ± 3.02% for liposome and 60.87 ± 2.54, 81.54 ± 3.24, 79.63 ± 3.14% for niosome). The least inhibition of inflammation was by BA free formula (39.13 ± 1.97, 70.77 ± 2.71, and 68.52 ± 2.37% at 1, 3, and 5 h, respectively). This study indicate that vesicular system was more effective than free extract probably due to the encapsulation of the extract inside the vesicle and their small size [139]. 

The bioavailability of phytochemicals has been greatly improved when formulated into phytosomes compared to free phytochemicals. Ju Ho et al. investigated the anti-inflammatory effect of *C. asiatica* phytosomes in a mouse model of phthalic anhydride-induced atopic dermatitis. They found that *C. asiatica* phytosomes successfully inhibited inflammatory activity by macrophage, which could be a promising tool for the management of atopic dermatitis [140]. *C. asiatica* extract contains different groups of phytoconstituents, such as siaticoside, asiatic acid and madecassic acid, and is known for its anti-inflammatory activity [141]. 

Phytosomes have been formulated in gel [130] and cream [122] for topical applications. Djekic et al. reported the formulation of a 18β-GA phytosomes loaded hydrogel by dispersion of 18β-GA phytosomes and carbomer with water. Then, formula was neutralized by 10% sodium hydroxide. Humectant was added later to form hydrogel. The study reported satisfactory physical stability during the first 30 days at room temperature and refrigerator with no organoleptic signs of change [130]. 18β-GA is a triterpenoid derivative extracted from *Glycyrrhiza glabra* which has anti-inflammatory, anti-irritant, and soothing effects [132,142,143]. Quercetin phytosomes cream is available in a cream form in the market (Quercevita^®^) which mainly contains lecithin, lecithin-quercetin, hydrogenated polydecene, glycerin and water [122].

In terms of scale up production of phytosomal formulations, the manufacturing process and the obstacles that could be faced should be recognized to ensure the successful transfer of phytosome technology from the laboratory to the market. One of the main advantages of phytosomes scale up process is that the materials required for phytosomes preparation are mainly safe which make phytosomes as good target to be synthesized in large scale for industrial production [144]. In addition, these materials were well evaluated in terms of toxicological effects which show low hazard report [144]. Moreover, the process of phytosome preparation is simple, does not required complicated and expensive instruments, and does not interfere with the encapsulated herbal substances as high chemical binding between phytosome’s phospholipid and phytochemical is occurred [144]. The easy process of scale up production of phytosomes from laboratory scale to industrial scale led to the successful reach of several phytosomal formulations to the market, and most of these products were developed by Indena [144,145]. For example, silybin phytosomes (Siliphos^®^) and curcumin phytosomes (Meriva^®^) are commercially available products currently used in cancer therapy [129]. Despite the easy scale up production of phytosomes, the high pH sensitivity of some phytosome components could limit the large scale synthesis of such phytosme-based formulations and should be considered during the manufacturing [146].

## 4. Conclusions and Future Prospects

Phytochemicals have potential for various therapeutic and aesthetic topical applications. Due to their low absorption profile, nanoengineered drug delivery systems are used to enhance the penetration of bioactive polyphenolic phytocompounds across biological barriers, thus increasing their bioavailability. One of these novel nanoplatforms is phytosomes nanocarriers. The lipid composition and nano-vesicular nature of phytosomes enables them to penetrate skin layers in a more effective manner than the extract of phytochemicals alone. Phytosomes are similar to liposomes in terms of vesicular structure and have a similar stability and skin penetration profile. However, in phytosomes, the phospholipid interacts with the phytochemicals via the formation of a H-bond between the phospholipid’s polar head and the polar functionalities of the bioactive components. This crucially enhances the stability and skin penetration of phytochemicals in comparison to liposomes. 

Many phytochemicals have been formulated successfully as phytosomes, and there are likely to be other phytochemicals that can benefit from phytosomes formulations. Future research might consider using phytosomes with other phytochemicals or the incorporation of drug and phytochemicals in the same nano-vesicle to produce synergistic effects.

## Figures and Tables

**Table 1:** Phytosome-based bioactive phytochemicals products available on the market.

Phytosome Formulation	Source Plant	Use	Reference
Silybin	*Silymarin marium*	Hepatoprotective and antioxidant activities	[73,74,75]
Ginkgo	*Ginkgo biloba*	Brain and vascular protection	[29,76]
Olive oil	*Europaea oil*	Anti-inflammatory, antioxidant, anti-hyperlipidemic activities and cardiovascular protection	[77]
Centella	*Centella asiatica*	Vein and skin disorders	[78]
Greenselect	*Camellia sinensis*	Antioxidant activity	[79]
Rutin	*Ruta graveolens* *Sophora japonica*	Rheumatoid arthritis	[80]
Curcumin	*Curcuma longa*	Hepatoprotective activity	[81,82]
Leucoselect	*Vitis vinifera*	Antioxidant activity	[28]
Ecdhinacea	*Echinacea augustifolia*	Immunomodulator	[83]
Cartaegus	*Cartaegus mexicana*	Antioxidant activity	[84]
Haw thorn	*Carteagus species*	Antihypertensive activity	[65]
Roscugenin	*Ruscus aculeatus*	Anti-inflammatory activity	[85]

**Table 2:** Phytosome-based formulations on clinical trials. All data were obtained from www.clinicaltrials.gov, accessed on 28 August 2021.

Phytosome Formulation	Condition	Clinical Trial Phase and No.	Sudy Outcome	Reference
Silybin	Prostate cancer	Phase II (NCT00487721)	High blood concentration of silybin	[87,88]
Silybin	EGFR mutant lung adenocarcinoma	Phase II (NCT02146118)	Under investigation	-
Green tea extract	Obesity	Phase IV (NCT02542449)	Maintaing weight following weight loss	[89]
Grape seeds extract	Early stages lung cancer	Phase II (NCT04515004)	Delay planned surgery of >14 days	-
Bergamot	Hypercholesterolemia	Not applicable (NCT04697121)	Anti-hypercholesterolemic activity	-
Quercetin	COVID-19	Phase III (NCT04578158)	Under investigation	-

**Table 3:** Key similarities and differences in the features of different lipid-based drug delivery systems that commonly used in the topical delivery of herbal substances.

Property	Phytosomes	Liposomes	Transferosomes	Reference
Structure	Lipid bilayer vesicles composed of different type of phospholipids that can chemically bound to phytochemicals	Lipid bilayer visecles composed of wider range of lipids including cationic, anionic, and neutral lipids	Lipid bilayer viscles composed of phospholipids, an edge activator (i.e., surfactant or bile salt ranging from 10–25%), low percentage of ethanol, and water as a vehicle	[55,91]
Encapsulation	The bioactive molecules are fixed by H-bond to the polar tip of the phospholipids	The active materials are incorporated in the aqueous core of the vesicles or in the lipid bilayer membrane	The active materials are incorporated in the aqueous core of the vesicles or in the lipid bilayer membrane	[55,97,98]
Preparation	The phosphatidylcholine and the phytochemicals actually form a 1:1 or 2:1 molecular complex that contains chemical bonds	The lipid compositions mixed alone, then the loaded materials added to the lipid thin film to form the complex with no chemical bonds formed	The lipid compositions mixed alone, then the loaded materials added to the lipid thin film to form the complex with no chemical bonds formed	[55,109,110]
Hydration buffer	Act with aprotic solvents such as acetone,1,4-dioxane, hexane, metyhlenechlorideand ethylacetate	Formed in the presence of a water or buffer solution	Formed in the presence of a water or buffer solution	[55,110]
Skin absorption	High skin absorption	Lower skin absorption	High skin absorption	[55,101]
Stability	High stability	Lower stability	High stability	[55,106]

**Table 4:** Examples of phytochemicals-phospholipids chemical interactions during phytosomes preparation.

Phytochemical	Type of Phytochemical	Type of Phospholipid	Chemical Interaction	Analysis Method	Reference
Quercetin	Polyphenols	PC	H-bonds with the polar group of the phosphplipid	1H-NMR, 31P-NMR, 13C-NMR	[127]
	Polyphenols	DPPC ^1^ (PC)	(1) Electrostatic interactions, (2) H-bonds with the polar group of the phosphplipid, (3) Hydrophobic interaction with fatty acyl chains	1H-NMR, 31P-NMR, 13C-NMR	[128]
Lycopene	Carotenoids (Terpenoid)	DPPC (PC)	Hydrophobic interaction with the acyl fatty acid chain	X-Ray	[133]
β-carotene, Lycopene	Carotenoids (Terpenoid)	POPC ^2^ (PC)	Hydrophobic interaction with the acyl fatty acid chain	X-Ray	[131]
Tyrosol, Verbascoside, Hydroxytyrosol	Polyphenols	PC	H-bonds with the polar group of the phosphplipid	1H-NMR, 31P-NMR, 13C-NMR	[33]
Saponin	Triterpene glycosides	PC	H-bonds with the polar group of the phosphplipid	1H-NMR, 31P-NMR, 13C-NMR	[15]
18-β-glycyrrhetinic Acid	Triterpenoids	Soy lecithin (PC)	H-bonds with the polar group of the phosphplipid	DSC	[132]

^1^ DPPC: Dipalmitoylphosphatidylcholine. ^2^ POPC: 1-palmitoyl-2-oleoyl-sn-glycero-3-phosphocholine.
